# Evidence for Multiple Distinct Interactions between Hepatitis B Virus P Protein and Its Cognate RNA Encapsidation Signal during Initiation of Reverse Transcription

**DOI:** 10.1371/journal.pone.0072798

**Published:** 2013-08-20

**Authors:** Hui Feng, Ping Chen, Fei Zhao, Michael Nassal, Kanghong Hu

**Affiliations:** 1 State Key Laboratory of Virology, Wuhan Institute of Virology, Chinese Academy of Sciences, Wuhan, China; 2 University Hospital Freiburg, Department of Internal Medicine II/Molecular Biology, Freiburg, Germany; 3 Biomedical Center, Hubei University of Technology, Wuhan, China; Drexel University College of Medicine, United States of America

## Abstract

Replication of hepatitis B virus (HBV) via protein-primed reverse transcription is initiated by binding of the viral P protein to the conserved ε stem-loop on the pregenomic (pg) RNA. This triggers encapsidation of the complex and the ε-templated synthesis of a short P protein-linked DNA oligonucleotide (priming) for subsequent minus-strand DNA extension. ε consists of a lower and upper stem, a bulge containing the priming template, and an apical loop. The nonhelical subelements are considered important for DNA synthesis and pgRNA packaging whereas the role of the upper stem is not well characterized. Priming itself could until recently not be addressed because in vitro generated HBV P - ε complexes showed no activity. Focussing on the four A residues at the base and tip of the upper ε stem and the two U residues in the loop we first investigated the impact of 24 mutations on viral DNA accumulation in transfected cells. While surprisingly many mutations were tolerated, further analyzing the negatively acting mutations, including in a new cell-free priming system, revealed divergent position-related impacts on pgRNA packaging, priming activity and possibly initiation site selection. This genetic separability implies that the ε RNA undergoes multiple distinct interactions with P protein as pgRNA encapsidation and replication initiation progress, and that the strict conservation of ε in nature may reflect its optimal adaptation to comply with all of them. The data further define the most attractive mutants for future studies, including as decoys for interference with HBV replication.

## Introduction

Chronic hepatitis B, caused by HBV, globally affects some 400 million people [1] and puts them at a high risk of progressing to liver cirrhosis and hepatocellular carcinoma (HCC) [2]. At present, only few and only partially effective therapies are available [2], [3]. Detailed elucidation of the HBV replication mechanism provides a chance to identify novel antiviral targets for therapeutic intervention [4]. One such potential target is the protein-primed reverse transcription mechanism [5] employed by HBV and the related animal hepadnaviruses, such as duck HBV (DHBV), to generate new DNA genomes.

**Figure 1:**
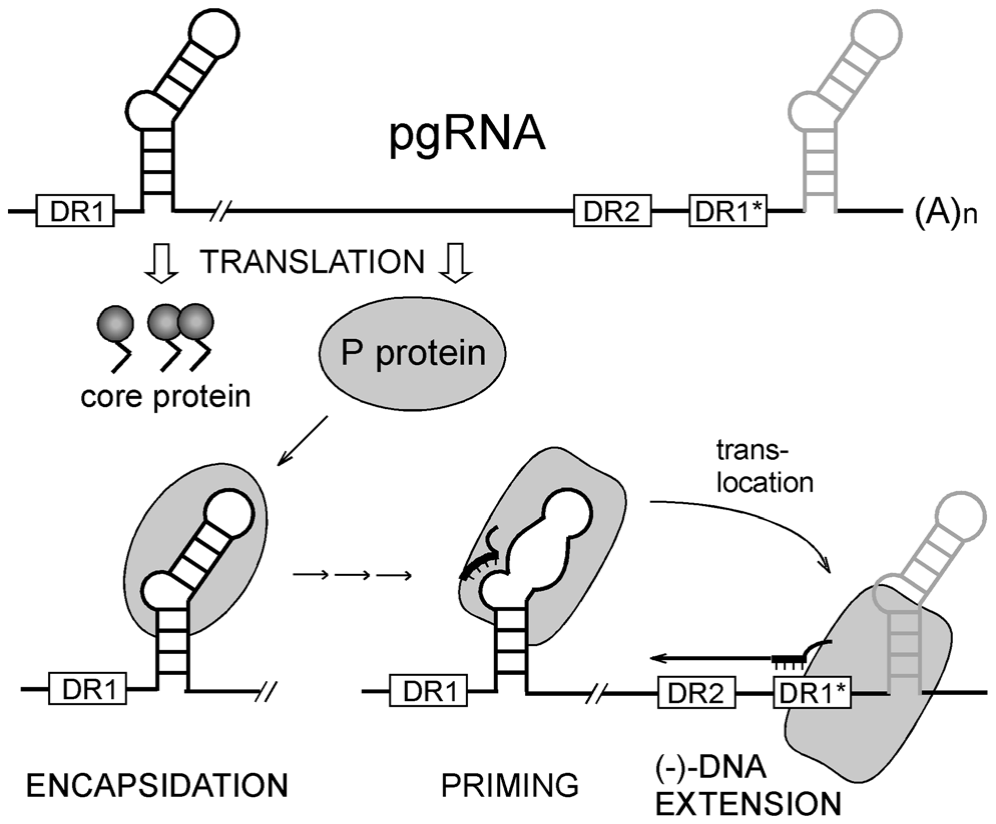
Key roles of the P protein - ε RNA interaction in HBV replication. The line with the hairpin structures represents the terminally redundant pgRNA which also serves as mRNA for core protein and P protein; ε and the direct repeats DR1, DR2 and DR1* are indicated. Binding of P to ε initiates their co-encapsidation, and also protein-primed reverse transcription. In this priming reaction, the 3′ nucleotide of the ε bulge and/or the first nucleotide of the upper stem (termed A1 in this study) template the covalent addition of the first DNA nucleotide to a Tyr residue in the TP domain (not explicitly shown) and its extension by two or three nucleotides along the bulge. Upon translocation to a matching acceptor site in DR1* the oligonucleotide is extended into full-length minus-strand DNA, with concomitant degradation of the pgRNA. Subsequent plus-strand synthesis (not shown) eventually yields the relaxed circular (RC) DNA found in virions. The differing shapes of ε and P symbolize conformational alterations that are as yet only well established for DHBV.

In all hepadnaviruses one of the viral transcripts, the pregenomic (pg) RNA, serves as template for reverse transcription within viral capsids (core particles) [6] (for reviews see [7], [8]). Co-packaging of pgRNA and the viral reverse transcriptase, called P protein, into newly forming capsids as well as replication are initiated by the specific interaction of P protein with the RNA encapsidation signal ε near the 5′-end of the pgRNA [9]; its overall structure, consisting of a lower and an upper stem, an internal bulge and an apical loop [10], [11], is similar in all hepadnaviruses [12]. All P proteins carry, beyond the evolutionarily conserved catalytic reverse transcriptase (RT) and RNase H (RH) domains, a unique Terminal Protein (TP) domain, separated from the RT domain by a dispensable spacer region. TP provides a specific Tyr residue (Y63 in HBV, Y96 in DHBV) as acceptor [13], [14], [15] for the first nucleotide of newly generated DNA ("protein-priming"). Binding to the cognate ε activates P for protein-priming, and the 3′ proximal half of the bulge [16], [17], for HBV possibly plus the following nucleotide [15], [18], provides the template for a 3- to 4- nt DNA oligo that is covalently linked to the Tyr residue in TP (Fig. 1). The P-linked DNA oligo is then translocated to a matching acceptor site at the 3′ proximal DR1* in the terminally redundant pgRNA from where it is extended into full-length (-)-strand DNA. Concomitant degradation of the pgRNA and subsequent (+)-strand DNA synthesis eventually yield the relaxed circular (RC) DNA typically found in virions (for reviews: [7], [8]).

Despite their overall similarity the ε stem-loops from HBV and DHBV are not functionally interchangeable. This and previous mutational studies indicate that the interaction between P protein and its cognate ε is highly specific and the result of both sequence- and structure-specific features in the RNA. Owing to the successful in vitro reconstitution of protein-priming for DHBV in rabbit reticulocyte lysate [19] or from purified components [20], [21], [22], such features are known in much more detail for DHBV than for human HBV where P - ε interaction studies have largely been restricted to mutational analyses of pgRNA encapsidation [10], [11] and DNA formation [18] in transfected cells. Collectively, the DHBV data suggest that the P - ε interaction during priming is a dynamic multi-step process in which the initial RNA binding is followed by conformational changes in both protein [23], [24], [25] and RNA [26]; these alterations are crucial to reach the priming-active state and require assistance by cellular chaperones [27], [28], [25], [22], although chaperone-dependence can be circumvented by employing truncated "miniP" proteins which lack the RH domain, the spacer and nonessential parts of the TP and RT domains but remain priming-competent [29], [30]. Most important within the DHBV ε RNA for achieving a priming-active complex are the central region comprising the bulge [31] and the apical loop, whereas in the upper stem various nt exchanges are tolerated [32], even in vivo [33]; in fact, part of the right half of the upper stem becomes expelled in priming-active P - ε complexes [26].

HBV P protein employed in analogous setups showed specific binding to ε RNA yet no priming activity [34]; in addition, binding did not require the upper stem and loop, although these subelements appear crucial for pgRNA packaging and DNA synthesis in cells.

Conversely, RNAs that bind to P protein but do not support virus replication lend themselves as decoys; these are structural mimics of functional RNAs that compete with the original RNA for the natural interaction partner(s) and thus can act as therapeutically useful inhibitors [35]). Particularly strongly binding RNA sequences (aptamers) may be selected from large sequence pools by appropriate SELEX (systematic evolution of ligands by exponential enrichment) procedures (for reviews: [36], [37]). To identify potential HBV ε decoys, we have recently performed such an in vitro SELEX screen using ε RNA libraries in which the entire upper stem, or the upper stem but not the 6 nt apical loop sequence, which according to NMR data forms a pseudo-triloop [38], [39], were randomized; selection was for binding to a recombinant HBV miniP protein [40]. In line with previous in vitro data [34], the selected RNAs displayed no preference for wild-type-like upper stem sequences but rather a general enrichment of A residues at the randomized positions. However, representative aptamers from the pool with maintained loop sequence displayed stronger competition with wild-type ε RNA than those from the completely randomized pool, implying that, at least in an unstructured A-rich upper stem framework, the loop sequence might contribute to P binding even in vitro. No inferences could be drawn on the role of base-pairing or nucleotide identity in the upper stem but according to previous reports [10], [41], [42], [43] numerous mutations in this region interfere with pgRNA packaging and/or replication. However, the near exclusive use of multiple simultaneous mutations hampered more detailed interpretations.

Based on these considerations and the strategic positions of selected residues within or close to non-paired regions (see below) we here focused on mutationally analyzing the functional role of six individual upper stem positions, namely all of the four A residues, i.e. A1 and A2 immediately above the bulge (numbers referring to positions within the upper stem), A9 and A10 close to the apical loop, and U13 and U15 within the pseudo-triloop. To address a potential impact of base-pairing, we also included U28 and U29 which can pair with A1 and A2 to close the bulge.

The corresponding mutations, in the context of a complete HBV genome, were first investigated for their effect on DNA synthesis in transfected human hepatoma cells. Next, in order to trace the underlying mechanism, those mutants displaying substantial defects in DNA accumulation were analyzed for pgRNA packaging. Finally, while this work was in progress, Jones et al. [44] reported a new cell-free system to directly address the HBV protein-priming reaction. This provided an opportunity to also look at the effect of selected mutations on the very first steps of HBV replication. As shown below, the combined results revealed very distinct impacts, correlating with the position of the respective mutations within ε, on divergent steps of HBV DNA synthesis, thus confirming the dynamic multi-step nature of the P - ε interaction as well as suggesting clues towards more effective ε decoys.

## Results

### Predicted location of the targeted positions in the HBV ε upper stem and mutant design

**Figure 2:**
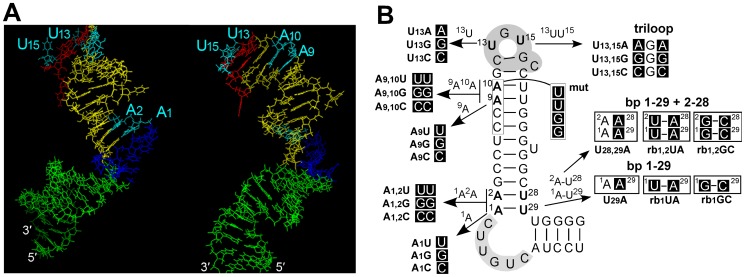
Predicted spacial location of the targeted upper stem residues and mutational design. (A) Model for the 3D structure of the ε stem-loop. 3D structure prediction as implemented in the MC-Fold and MC-Sym program package [46] was combined with NMR-based structural information on the upper stem and apical loop ([39], PDB entry 2IXY) to derive a tentative model for the entire ε element with its lower stem (green), bulge (blue), upper stem (yellow) and apical loop (red). The same model is shown from two different angles to provide a visual impression of the spacial distribution of the targeted residues A1, A2, A9, A10, U13 and U15 (all in cyan). Their major groove location and close juxtaposition to the bulge and loop, respectively, is supported by the NMR data; other features, including the relative orientation of upper stem vs. lower stem, are arbitrary. (B) Specific mutations investigated. Nucleotide exchanges and their positions in a 2D representation of ε together with the designations of the mutants are indicated. A linear representation is shown in Table 1.

RNA loops and bulges as well as their distorted connections into regular double-helical regions provide a rich repertoire of distinct structural features predestining them as specific recognition elements, often by opening the major groove [45]. Since simple 2D structure representations do not reveal such features, we first combined published NMR 3D structure data for the (isolated) upper stem of HBV ε [39] and structure prediction to model [46] the entire stem-loop and visualize the location of the 6 residues in question. As shown in Fig. 2A, the A residues are at or close to the junctions between double-helical sections and the single-stranded bulge and the pseudo-triloop which harbors U13 and U15; the A residues face the major groove in the double-helical portion, as do U13 and U15 in the pseudo-triloop. Hence the upper stem A- and apical loop U-residues would be suitably positioned to contribute to the P - ε interaction.

Next we designed a set of 24 mutants, as summarized in Fig. 2B. A1, A9 and U13 were individually mutated to all other three nucleotides, A10 and U15 in various combinations with mutations at A9 or U13, respectively. To assess the importance of A1 and A2 being base-paired with U28 and U29 we also introduced mutations at the latter positions, thus replacing the original basepairs by others, or preventing basepair formation; mutants in which the first or first plus second base-pair above the bulge is restored were named rb1NN and rb1,2NN, with NN indicating the specific new base-pair. Lastly, we included a mutant (termed "mut" below) in which the four consecutive nucleotides C_7_C_8_A_9_A_10_ were replaced by GGUU (Fig. 2B); while reportedly reducing but not ablating RNA packaging (mutant "upperL2" in [41]) the same mutations completely abrogated in vitro P binding (mutant "U-L-U" in [34]); this variant was therefore used as negative control. All mutations were introduced into the 5′ copy of ε encoded in the 1.1× unit length wild-type HBV genome present in the CMV-IE promoter-driven expression vector pCH-9/3091 [47].

### Differential impact of mutations of the upper stem adenines and loop uracils on HBV replication

**Figure 3:**
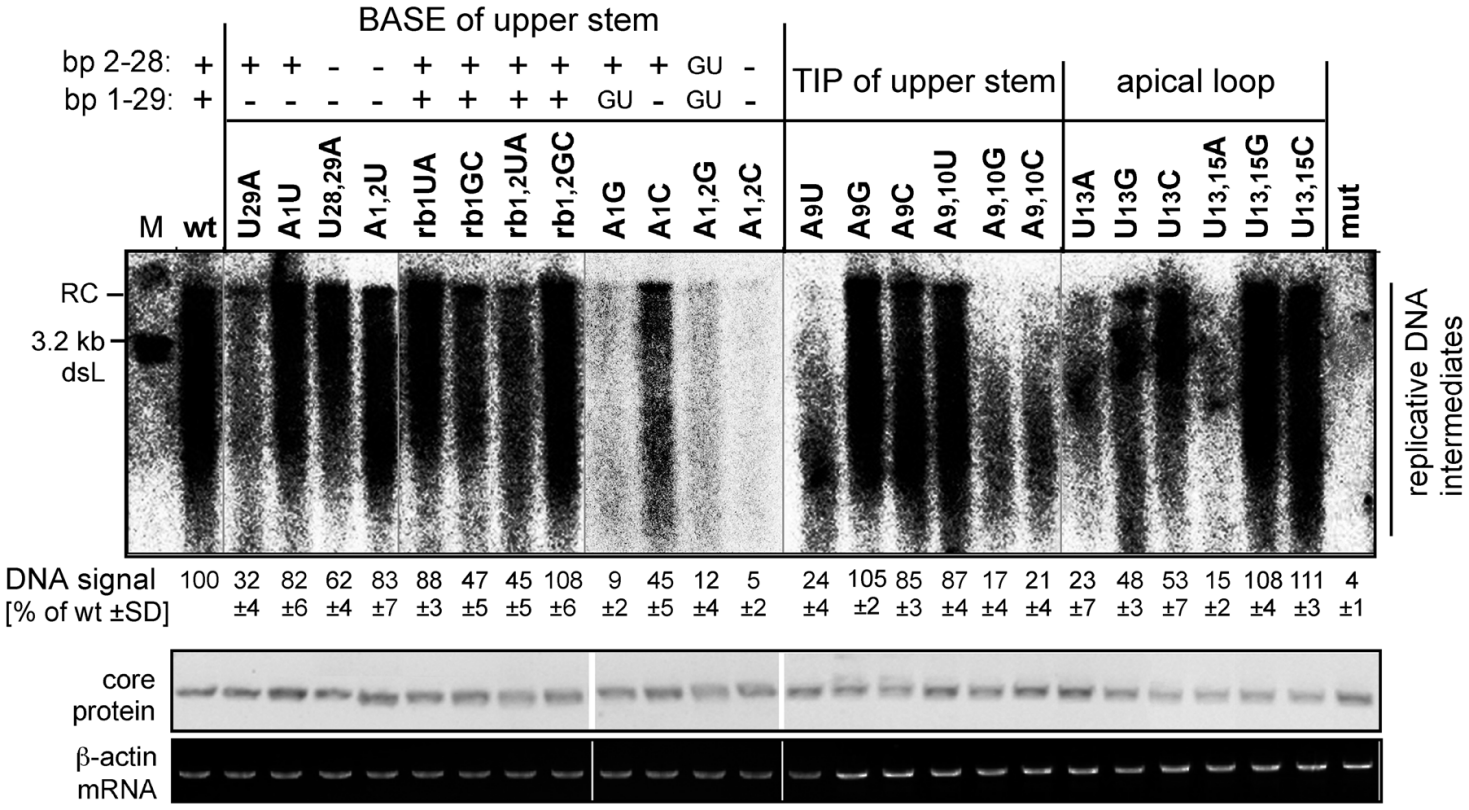
Impact of individual ε mutations on viral DNA accumulation. HepG2 cells were transfected with the wild-type (wt) HBV expression vector pCH-9/3091, or derivatives containing the mutant 5′ ε sequences shown in Fig. 2B. The + or − signs indicate whether canonical base-pairs could form between residues at the A1-U29 and A2-U28 positions; potential G-U pairs are separately indicated. Viral DNAs from cytoplasmic nucleocapsids were monitored by Southern blotting (top panel) using a ^32^P-labeled HBV DNA probe; M, 3.2 kb restriction fragment corresponding to a unit length double-stranded linear (dsL) HBV genome. As controls, core protein and β-actin mRNA levels in the source lysates were monitored by Western blotting (middle panel) and RT-PCR (lower panel). Numbers below each lane show the accumulation of viral DNA replicative intermediates, measured by phosphorimaging, relative to those produced by the wild-type HBV construct which was set to 100. Mean values ± standard deviation were derived from two independent experiments.

Formation of HBV replicative DNA intermediates indicates that all prior steps have successfully been passed, including expression of core and P protein, assembly of pgRNA plus P containing nucleocapsids, and protein-primed initiation and extension of minus-strand DNA synthesis. We therefore transfected the HBV expression vectors for the 24 ε upper stem variants, plus the parental wild-type HBV vector as positive control and the P protein binding-deficient ε mut vector as negative control, into HepG2 cells; four days later viral DNAs associated with cytoplasmic nucleocapsids were analyzed by Southern blotting, using a ^32^P-labeled DNA probe covering the whole HBV genome (Fig. 3). As controls for core protein expression and equal loading, aliquots from the same cytoplasmic lysates were analyzed by Western blotting (panel labeled "core") and RT-PCR for β-actin mRNA (panel labeled β-actin mRNA).

Compared to the replicative DNA intermediates generated by the wild-type HBV vector (lane wt), the mutants displayed distinct classes of phenotypes (Fig. 3). Some mutants showed wild-type-like patterns and amounts of DNA (A1U; A1,2U; rb1UA; rb1,2GC; A9G; A9C; A9,10U; U13,15G; U13,15C), some others modest reductions (down to ∼30% of the wild-type) in DNA amounts (rb1GC; U29A; rb1,2UA; U28,29A; A1C; U13G; U13C). The remaining variants displayed severe reductions in DNA amounts (<25% of the wild-type); however some still produced a wild-type-like pattern of apparently full-length DNA (A1 and A1+A2 mutants A1G, A1,2G, A1,2C) while for the others mainly faster migrating but no full-length products were detected (A9, A9+A10, and some U13+U15 mutants). This would be compatible with the former mutations largely affecting efficiency of protein-primed initiation whereas the latter appeared to also impact the quality of minus-strand and possibly plus-strand DNA extension. For the P binding-defective mut variant no signals were detected. The comparable RT-PCR signals for β-actin mRNA indicated that the source lysates were derived from similar numbers of cells. The levels of Western blot detectable core protein varied by less than two-fold and thus could neither account for the massive differences in DNA levels; for instance, the core protein signals for the low DNA samples A1G or A1,2C or the negative control variant mut were not weaker than those for samples showing high DNA levels such as wild-type HBV or mutants rb1UA or rb1,2GC.

**Figure 4:**
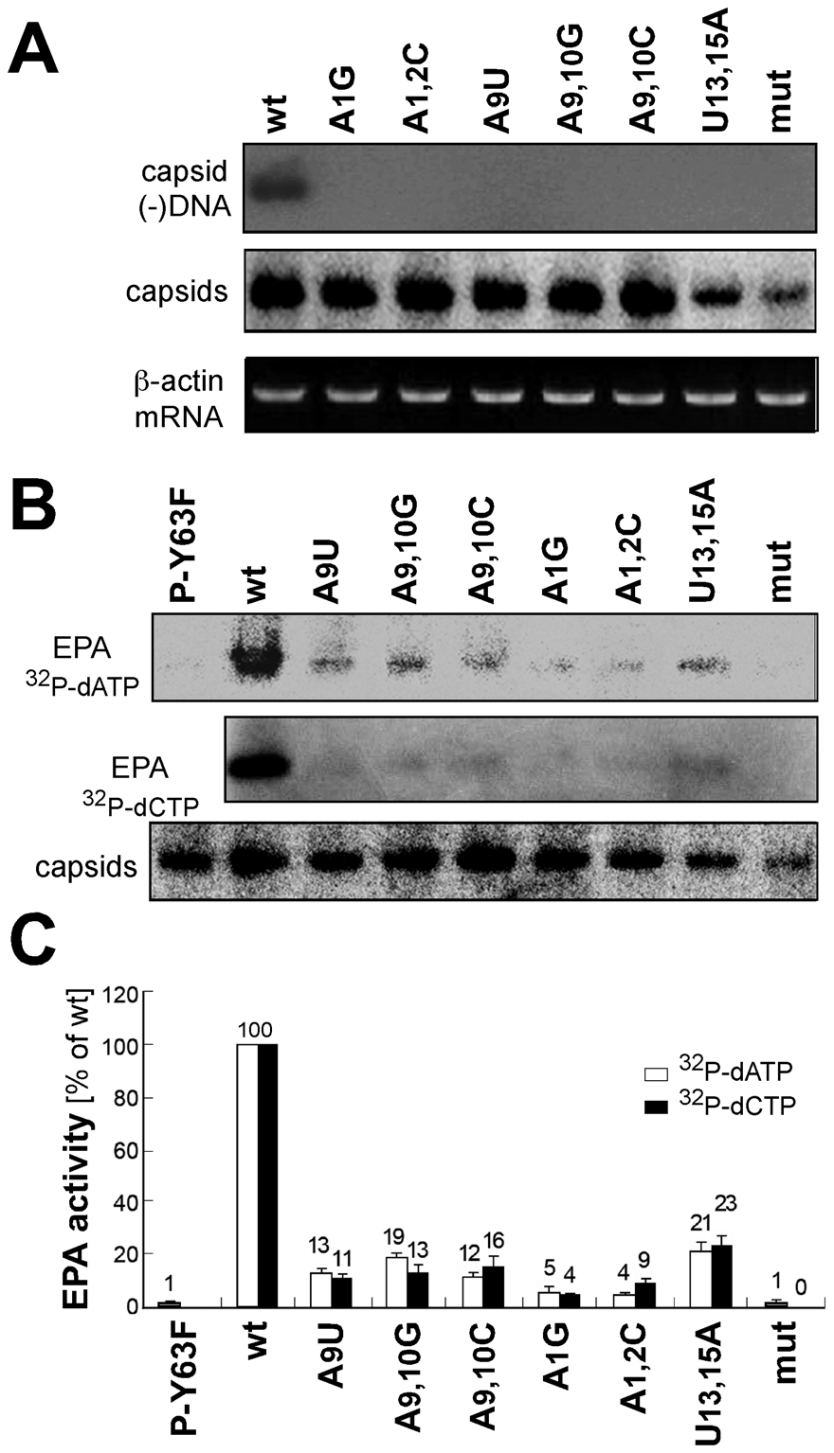
Direct confirmation of low DNA content in intact capsids derived from mutant ε constructs. (A) DNA detection by molecular hybridization. Cytoplasmic capsids from cells transfected with the indicated constructs were separated by NAGE. After blotting, HBV DNA in the capsids was monitored by hybridization with a minus-strand specific probe, and capsids by immunodetection (panel labeled capsids). β-actin mRNA as determined by RT-PCR (panel labeled β-actin) served as loading control. (B) Endogenous polymerase assays (EPAs). One aliquot each of cytoplasmic capsids was subjected to EPA conditions in the presence of α-^32^P-dATP or α-^32^P-dCTP, then separated by NAGE. Labeled products associated with the capsids were visualized by autoradiography. Y63F refers to a replication-defective HBV construct in which the priming Tyr63 residue of P was replaced by Phe. A third aliquot from each sample was used for immunodetection of NAGE-separated capsids (panel labeled capsids). (C) Relative EPA activities. The bar graph shows the signal intensities generated by individual mutants for α-^32^P-dCTP and α-^32^P-dATP EPAs relative to that produced by wild-type HBV which was set at 100. Numbers are mean values from at least two independent experiments; error bars indicate standard deviation.

To directly confirm a low DNA content of the capsids produced from the mutants giving low Southern blot signals we employed native agarose gel electrophoresis (NAGE) in which intact capsids are analyzed. First, capsids in cytoplasmic lysates from cells transfected with the indicated constructs were separated by NAGE and, after blotting, were subjected to immunodetection of capsids or molecular hybridization with a (+)-polarity probe specific for HBV (−)-DNA (genome positions 837–1284). As shown in Fig. 4A, all samples contained capsids but only those from the wild-type HBV transfected cells gave a comigrating distinct DNA signal. The lower sensitivity of this assay compared to the Southern blot likely relates, apart from the shorter size of the single-stranded probe, to the fact that in the NAGE assay much smaller fractions of cytoplasmic lysate are employed than were used to isolate capsid-associated DNA for Southern blotting. Clearly, however, the mutant-derived capsids contained much less DNA than wild-type HBV capsids. As a more sensitive test, we next used the endogenous polymerase assay (EPA) in which the capsid-associated P protein extends immature DNAs upon provision of exogenously added dNTPs, one of which is ^32^P labeled. To this end, one aliquot each of the respective cytoplasmic lysates was separated by NAGE for anti-capsid immunoblotting, two other aliquots were prior to NAGE incubated with either α-^32^P-dATP or α-^32^P-dCTP plus the lacking three other dNTPs. A replication-defective P protein mutant in which the protein-priming Tyr63 residue was replaced by Phe (Y63F) was included as further negative control in some experiments. As shown in Fig. 4B, all samples contained capsids, and both α-^32^P-dCTP and α-^32^P-dATP labeled EPA assays gave comparable results; wild-type capsids yielded strong signals, the mut and Y63F variants no signal, and the signals from the respective mutants, quantitated by phosphorimaging, correlated well with those obtained by Southern blotting (Fig. 4C).

Together these data documented that mutations at the A1, A2, A9, A10 positions in the upper stem and at the U13 and U15 positions in the apical loop can, but do not necessarily have to, negatively affect viral DNA accumulation in capsids. While a more detailed interpretation is provided in the Discussion, we note that canonical Watson-Crick base-pairing of the residues at the A1/A2 positions immediately following the bulge is not required for efficient DNA synthesis, as shown by several wild-type-like behaving mutants in which the natural A1+A2 pairing with U28+U29 was absent (e.g. A1U; U28,29A). Similarly, there was no direct correlation between DNA synthesis and the presence of the natural A9+A10 pairing with U19/U20 (e.g. A9C; A9,10U). However, low levels of viral DNA could not only relate to impaired DNA synthesis but also defects in encapsidation of the pgRNA template.

### Differential impact of upper stem versus apical loop mutations on pgRNA encapsidation

**Figure 5:**
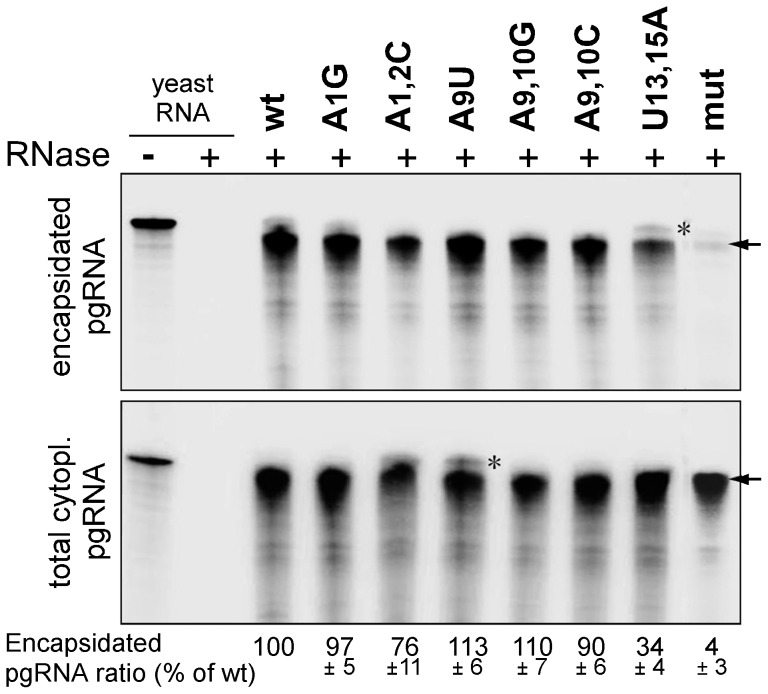
Impact on pgRNA encapsidation of selected mutants displaying reduced DNA accumulation. Total cytoyplasmic RNA and capsid-associated RNA from cells transfected with the indicated constructs were analyzed by RNase protection assays via hybridization to a 314 nucleotide antisense riboprobe (indicated by an asterisk) containing 41 nucleotides of non-HBV sequence; RNase digestion is expected to yield a protected fragment of about 270 nt (indicated by the arrow). Numbers below each lane show the encapsidation efficiency, measured as the ratio of encapsidated versus total pgRNA, relative to that produced by the wild-type HBV construct which was set to 100. Mean values ± standard deviation are from three independent experiments.

Based on the DNA analyses (Fig. 3 and Fig. 4), we next analyzed variants with strongly reduced viral DNA levels for pgRNA encapsidation; wild-type HBV and the mut variant served as references. To this end, we employed RNase protection assays to compare the levels of pgRNA contained in cytoplasmic nucleocapsids versus in total cytoplasmic RNA preparations. As shown in Fig. 5, wild-type HBV-like amounts of pgRNA were detected in all total RNA preparations and also in most capsid-associated RNA samples, with two exceptions; the P binding defective mut variant showed no signal and the apical loop double-mutant U13,15A a substantially (by two thirds) weakened signal. This was confirmed by semiquantitatively, via phosphorimaging, relating the signal intensities of capsid RNA versus total RNA in each sample. Accordingly, the apical loop double mutant was the only one for which the low levels of detectable viral DNA (Fig. 3 and Fig. 4) correlated with substantially reduced pgRNA packaging; however, the defect in DNA synthesis appeared more pronounced. For the A1/A2 and A9/A10 mutants, in contrast, factors other than impaired pgRNA encapsidation must be responsible for the severely reduced DNA accumulation.

### Assessment of upper-stem and apical loop mutants as protein-priming templates

**Figure 6:**
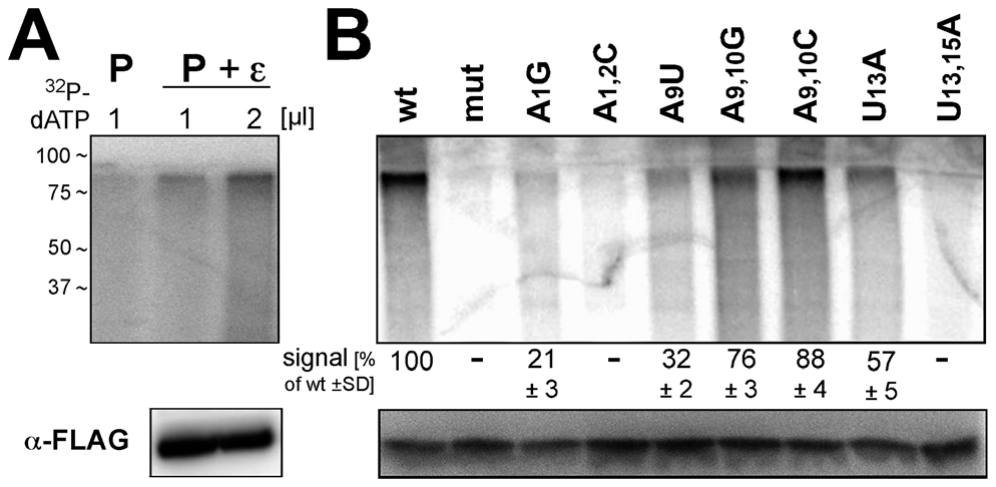
In vitro priming activities of selected ε RNAs showing reduced DNA accumulation in cells. (A) Increased priming signals by co-expression of wild-type ε RNA and increased α-^32^P-dATP concentration. FLAG-tagged HBV P protein from cells transfected with only the P protein vector (lane P), or cotransfected with a wild-type ε RNA expression vector (lanes P + ε) was immobilized on anti-FLAG antibody beads. One third each of the immunoprecipitate was incubated with 1 µl or 2 µl of α-^32^P-dATP (3,000 Ci/mmol) as reported [44]; subsequently, the beads were boiled in SDS-PAGE sample buffer, and the released material was analyzed by SDS-PAGE and autoradiography. The remaining one third of the immunopellet was analyzed for FLAG-tagged P protein by Western blotting (panel anti-FLAG). (B) In vitro priming activities of selected ε RNA variants. FLAG-tagged P protein complexes with the indicated ε RNAs were expressed, affinity purified and subjected to in vitro priming conditions as in (A), using 2 µl α-^32^P-dATP. The molecular mass marker positions indicated on the left (in kDa) are approximations inferred from the respective marker protein positions on the SDS-PAGE gels used for the anti-FLAG immunoblots which were run in parallel under identical conditions. Numbers below the autoradiogram indicate mean signal intensities ± standard deviation from two independent experiments relative to that produced by the wild-type ε RNA complex which was set to 100.

The identification of several ε mutants that did not accumulate viral DNA despite intact pgRNA encapsidation suggested that their defects related to impaired protein-priming, or impaired primer translocation to DR1*, preventing generation of full-length minus-strand and eventually RC-DNA. Until recently, HBV protein-priming could not be addressed in vitro. Jones et al. [44] have now partly overcome this problem by affinity enrichment of FLAG-tagged P protein - ε RNA complexes (plus associated cell factors) from HEK293T cells co-transfected with P protein plus ε RNA expression vectors. The immobilized complexes were capable of in vitro protein-priming when supplied with dNTPs, as detectable by covalent radiolabeling of P protein when α-^32^P-dNTPs are used. We therefore applied an analogous system to address the priming competence of various mutants that had shown severe defects in DNA accumulation. As a first test, we compared P protein expressed in the absence or presence of co-expressed wild-type ε RNA. As shown in Fig. 6A, clearly detectable P protein labeling upon provision of α-^32^P-dATP required ε RNA co-expression as reported [44], and the signal was enhanced by doubling the amount of α-^32^P-dATP (lanes P + ε vs. lane P). The presence of P protein was confirmed by anti-FLAG immuno blotting. Next, analogous assays were performed with complexes from cells co-transfected with the P protein expression vector plus one each of the vectors for the indicated eight mutant ε RNAs. As shown in Fig. 6B, the wild-type RNA but not the P binding-defective mut variant produced a clear signal whereas for the mutants a wide range of signal intensities was seen. Signals for the double mutants A1,2C and U13,15A were too weak for quantification. The single A1 and A9 mutants A1G and A9U showed weak but distinct bands; band intensity was increased for the U13 mutant U13A and almost reached wild-type levels for the A9/10 double mutants A9,10G and A9,10C; these differences were not due to different amounts of P protein, as shown by anti-FLAG immunoblotting (Fig. 6B, bottom panel).

Assuming that the quality of the P protein in the nine different preparations was comparable (because ε RNA added after complex isolation from the cells is not accepted as template [44], individual preparations are required), and if the results obtained with dATP also apply to the other dNTPs, these data suggest that failure of DNA accumulation for the A1/A2 and U13/U15 mutants results from a general impairment of protein-priming, whereas the A9/A10 mutations and the U13A mutation impede a step following addition of the first nucleotide but preceding primer translocation to the proper acceptor site at DR1* (see Fig. 1, and Discussion); as we have not directly determined RNA encapsidation efficiency for the U13A variant, a limited contribution of reduced RNA packaging to lowered DNA accumulation is currently not excluded.

Altogether, these data revealed that seemingly minor changes in the ε sequence can have more differentiated impacts on the different functional aspects of the HBV P protein - ε interaction than suggested by previous studies.

## Discussion

In this study we have systematically investigated the impact of mutations at six strategically located positions in the upper stem and apical loop of the HBV ε signal with respect to viral DNA accumulation, pgRNA packaging, and in vitro priming activity. The individual mutants and their functional phenotypes are summarized in Table 1. The data revealed that seemingly minor changes in sequence caused several divergent replication phenotypes, ranging from virtually no impact over modest to massive reductions in viral DNA accumulation; as discussed below, these defects in DNA synthesis correlated with distinct defects in the preceding steps that depend on a productive P protein - ε RNA interaction.

**Table 1:** Sequences of investigated ε mutants and summary of phenotypes.

Designation	upper stem (left)	loop	upper stem (right)	a)DNA accumulation	a)EPA activity	^b)^Full-length (FL) or Short (S) DNA	a)pgRNA packaging	a)in vitro priming (dATP)
wt	AAGCCUCCAAG	CUGUGC	CUUGGGUGGCUU	+++	+++	FL	+++	+++
A1U	U..........	......	............	+++	nd	FL	nd	nd
A1G	G..........	......	............	+/−	+/−	FL	+++	+/−
A1C	C..........	......	............	++	nd	FL	nd	nd
A1,2U	UU.........	......	............	+++	nd	FL	nd	nd
A1,2G	GG.........	......	............	+/−	nd	FL	nd	nd
A1,2C	CC.........	......	............	+/−	+/−	FL	+++	–
U29A	...........	......	...........A	+	nd	FL	nd	nd
U28,29A	...........	......	..........AA	++	nd	FL	nd	nd
rb1UA	U..........	......	...........A	+++	nd	FL	nd	nd
rb1GC	G..........	......	...........C	++	nd	FL	nd	nd
rb1,2UA	UU.........	......	..........AA	++	nd	FL	nd	nd
rb1,2GC	GG.........	......	..........CC	+++	nd	FL	nd	nd
A9U	........U..	......	............	+	+	S	+++	+
A9G	........G..	......	............	+++	nd	FL	nd	nd
A9C	........C..	......	............	+++	nd	FL	nd	nd
A9,10U	........UU.	......	............	+++	nd	FL	nd	nd
A9,10G	........GG.	......	............	+/−	+/−	S	+++	++
A9,10C	........CC.	......	............	+/−	+/−	S	+++	+++
U13A	...........	.A....	............	+/−	nd	(FL+) S	nd	++
U13G	...........	.G....	............	++	nd	FL	nd	nd
U13C	...........	.C....	............	++	nd	FL	nd	nd
U13,15A	...........	.A.A..	............	+/−	+/−	(FL+) S	+	–
U13,15G	...........	.G.G..	............	+++	nd	FL	nd	nd
U13,15C	...........	.C.C..	............	+++	nd	FL	nd	nd
mut	......GGUU.	......	............	–	–	none	+/−	–

a) Values are categorized relative to wild-type HBV (100%) as follows: +++, 66–100%; ++, 33–65%; +, 25–32%; +/−, 10–24%; −, not detectable; nd, not determined. ^b)^ Derived by visual inspection of the autoradiograms shown in Fig. 3.

### Tolerated upper stem mutations

In natural HBV isolates, the ε sequence is one of the most highly conserved regions [48], [49], as confirmed in a recent ultra-deep sequencing study [50]. Mutations that affect the ε structure, e.g. stop codons in the overlapping preC ORF which prevent precore translation and HBeAg formation, are almost invariably accompanied by additional compensatory mutations that restore the genuine stem-loop architecture. The HBV ε sequence is also largely conserved in the other mammalian hepadnaviruses; in contrast, especially the upper stem is much more variable between avian hepadnaviruses [32], and DHBVs with various mutations in the upper ε stem are viable even in ducks [33]. Hence a reason for the strict conservation of ε amongst the mammalian viruses is not obvious. The ten HBV ε mutants from our study which showed no substantial defect in DNA accumulation indicate that the authentic ε sequence is not a prerequisite for productive interactions with P protein; notably, tolerated mutations were found at all investigated positions, including the structured apical loop [38], [39]. Hence natural sequence conservation may be driven by minor differences in replication efficiency that come to bear only upon continuous genome propagation in vivo but not in single round transfection studies [33]. Candidate factors beyond the immediate P - ε interaction might be the overlapping preC ORF and its translation products, or cis-elements that act after the initial priming step. For instance, base-pairing between the left half-stem of ε and the downstream φ element appears to contribute to replication efficiency [51], [52], [53]. However, the A1 and U13/U15 positions mutated are not, and the A2 and A9/A10 positions are only peripherally involved in this base-pairing (Fig. S1), making a substantial contribution to the poor DNA accumulation of mutants such as A9,10G or A9,10C unlikely.

### Impact of the A1/A2 positions and their involvement in base-pairing

Residues A1 and A2 immediately follow the template region in the ε bulge, and A1 [15], [18] may as well as the preceding C residue [44] serve as initiation template for the DNA oligonucleotide primer (Fig. 1); hence A1 and A2 would be expected to most directly affect protein-priming. Indeed, the single A1G exchange caused a drastic reduction in DNA accumulation (Fig. 3, Fig. 4) and gave an extremely weak in vitro priming signal with ^32^P-dATP (Fig. 6) although it did not negatively impact pgRNA encapsidation (Fig. 5). In contrast, the analogous A1C exchange had only a modest, and the A1U mutation no detectable influence on DNA accumulation. The corresponding A1/A2 double mutations to GG and UU (A1,2G and A1,2C) showed the same phenotypes as the single G and U mutations, whereas the A1/A2 CC double exchange strongly enhanced the negative impact of the single A1C mutation, both in DNA accumulation (Fig. 3) and ^32^P-dATP priming (Fig. 6); however, it also did not interfere with pgRNA encapsidation (Fig. 5), i.e. a packaging-proficient interaction with P was maintained.

One possibility to explain these different phenotypes was the importance of the base-pairs closing the ε bulge. For instance, preventing A-U pair formation by combining the authentic A1 or A1 plus A2 residues with A substitutions at the opposite U28 and U29 positions (U29A; U28,29A) reduced DNA accumulation similarly as the A1C exchange (Fig. 3) although the sequence of the template region was preserved; conversely, the strong replication defects caused by the A1G and A1A2>GG (A1,2G) mutations were partially or even completely rescued by replacing U29 (rb1GC) or U28 and U29 (rb1,2GC) by C residues.

However, the other mutants suggest a more complex correlation. Mutants containing U residues at A1 or A1+A2 (A1U; A1,2U) displayed wild-type-like DNA accumulation although base-pairing with U28/U29 was prevented (Fig. 3). Replacing the original A1-U29 basepair by a U-A pair (rb1UA) had no negative effect but replacing both the A1-U29 and A2-U28 pair by U-A (rb1,2UA) reduced DNA accumulation.

**Figure 7:**
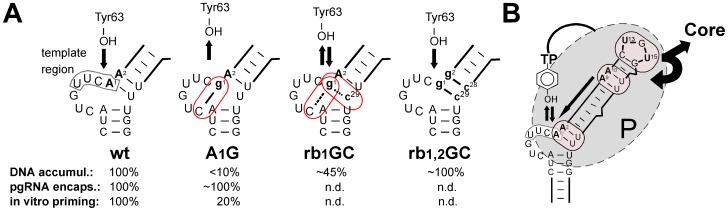
Models for the position-related functional impacts of mutations in the upper stem. (A) Base-pairing at the A1/A2 - U28/U29 positions may indirectly affect formation of a priming-active template structure. Replacement by G of A1 (A1G) or A1 plus A2 (A1,2G; not shown) nearly abrogated in vitro priming and DNA accumulation, whereas DNA accumulation was largely restored via concomitant replacement by C of U29 (rb1GC) or U28 plus U29 (rb1,2GC); n.d., not determined. Sequence-dependent formation of non-productive alternative structures could explain these divergent phenotypes. The model implies a (not experimentally proven) cross-bulge pair between A1g and the 5′ terminal bulge C residue (highlighted by an oval) which impairs template utilization (upward pointing arrow). This would be disfavored by stabilizing the original base-pairing pattern, via one (rb1GC; equally favored g1-C or g1-U29 pairs) or better two (rb1,2GC; mostly g1-C29 plus g2-C28) pairs at the base of the upper stem. U residues at the A1 and A2 position (A1U; A1,2U) cannot form cross-bulge base-pairs (not shown) and do not induce defects in DNA accumulation. See text for further details. (B) Major position-related functional impacts of upper stem mutations. This conceptual model summarizes the divergent phenotypes of mutants with a negative impact on viral DNA accumulation. Negatively acting mutations at A1/A2 had no defect in pgRNA encapsidation, produced a wild-type-like pattern of DNA, however at greatly reduced levels, and showed low or no in vitro priming. This suggests a major impact on priming efficiency per se (symbolized by the upward and downward arrows). Negatively acting mutations at A9/A10 did also not interfere with pgRNA packaging; however, only faster migrating DNA species were formed. Together with their clear in vitro priming activity, this implies a defect in primer translocation to the DR1* acceptor, e.g. via formation of an improper primer by improper initiation site selection (symbolized by the arrow pointing towards the template region). Negatively acting apical loop mutations reduced pgRNA encapsidation, displayed overall reduced DNA accumulation plus an excess of faster-migrating over full-length DNA, and showed low in vitro priming. This implies a general impact on P binding and/or formation of an encapsidation-proficient structure (symbolized by the curved arrows pointing towards P and core), combined with partial defects in protein priming efficiency and proper initiation site selection. See text for further details.

These different data sets may be reconciled by the negatively acting mutations disturbing, to different extents, formation of a priming-compatible structure of the template region in the bulge by inducing non-productive alternative structures (Fig. 7A). For instance, a G at the A1 position could pair with the 5′ terminal C of the bulge at the cost of the weak A1G-U29 pair; such an improper cross-bulge base-pairing would be partially counteracted by keeping A1G paired within the upper stem by the U29C mutation (rb1GC), and to an even larger extent in the quadrupel mutant A1A2>GG plus U28U29>CC (rb1,2GC). A similar case can be made for improper base-pairing of the A1C and A1,2C mutants to the G residue at the third bulge position. Conversely, with U residues at the A1 and A2 positions no inappropriate cross-bulge base-pairings could occur for lack of A residues in the bulge. Obviously, the current data do not yet prove this model but they provide a conceptual framework for future experiments; particularly revealing should be a direct structural comparison of the corresponding RNAs in their free and P protein-bound state, as previously applied to the DHBV P protein - ε RNA interaction [26].

### Impact of the A9/A10 positions at the tip of the upper stem

The A9 and A9+A10 mutants also presented with complex phenotypic DNA patterns. Single replacements of A9 by G (A9G) or C (A9C) were completely tolerated, replacement by U (A9U) was not; in contrast, concomitant substitution of A10 by U (A9,10U) restored function of the A9U mutant whereas two consecutive G (A9,10G) or C (A9,10C) mutations nearly ablated DNA accumulation. Hence the functional impact of individual mutations is highly context-dependent; rather than the mere number of base-pairs that context appears to define whether or not individual nucleotides will be available for productive interactions with P protein or not. Again, elucidating the structures of the free versus P protein-bound RNAs should provide new insights, yet even with the new in vitro priming system this will remain a difficult task because the ε RNA in the P protein - RNA complexes isolated from the transfected cells can not be exchanged in vitro [44].

However, already our current data imply that the mechanism by which A9/A10 mutations interfere with DNA accumulation differs from that exerted by the A1/A2 mutations. For the latter mutants, the reduced DNA signals in the Southern blot still extended up to the full-length position (Fig. 3, A1G, A1,2G, A1U) whereas for the A9/A10 mutants the bulk of products migrated faster, with little or no signal at the full-length position (Fig. 3, A9U, A9,10G, A9,10C). Furthermore, the corresponding A9/A10 mutants displayed modest (A9U) or even strong (A9,10G; A9,10C) in vitro priming activity (Fig. 6) whereas very low or no signals were seen for the A1/A2 mutants A1C and A1,2C (Fig. 6). An interpretation in line with these data (Fig. 7B) is that mutations at A1/A2 interfere with protein-priming per se, whereas A9/A10 mutations allow priming but negatively impact subsequent primer translocation to DR1* and/or minus-strand DNA extension. One scenario is that A9/A10 mutations affect initiation site selection, such that the sequence or length of the protein-primed DNA oligonucleotide are incompatible with the genuine DR1* acceptor; as previously shown, non-DR1* matching primers can be translocated to alternative acceptor sites [18], [52], preventing full-length minus-strand DNA and subsequent RC-DNA synthesis. Another scenario results from the requirement for minus-strand DNA extension to replace ε as template by the pgRNA sequence preceding the DR1* acceptor. Hence mutations in ε that enhance binding to P may impede this template replacement. Again the current data do not allow firm mechanistic conclusions but they define a specific set of mutants for future more thorough investigation, in particular definition of the exact DNA sequences that are linked to P protein in the faster migrating products.

### Impact of the U residues in the apical loop

Earlier studies indicated that multiple simultaneous loop mutations severely affected DNA accumulation, mostly via impeding pgRNA encapsidation [41], [42], [18]. Single site mutations, such as variants L1 to L6 (named according to the position in the CUGUGC loop sequence [41]; U13 and U15 in our numbering correspond to L2 and L4) produced differing phenotypes, ranging from virtually no impact (L1, L2 and L6) to a substantial reduction (L4 and L5) or complete loss (L3) of encapsidation competence (measured using fusions of the ε sequence to heterologous reporter RNAs). However, only one specific replacement for each loop position was analyzed. In principal accordance, we found an only modest impact on DNA accumulation of replacing U13 by A (U13A, identical to the reported L2 mutation in [41]), and even less so by G or C substitutions (U13G and U13C). The U13A mutant also displayed substantial α-^32^P-dATP in vitro priming activity (Fig. 6). The strongest negative impact on DNA accumulation was seen for the U13/U15 double A mutation (U13,15A), which also yielded no detectable in vitro priming signal (Fig. 6). Moreover, this was the only of the tested variants with clearly reduced pgRNA encapsidation capacity (Fig. 5), suggesting a generally reduced ability to interact with P protein.

More surprising is the only modest or even lacking impact on DNA accumulation exerted by the U13G and U13C variants and the U13,15G and U13,15C double mutants; based on simple Watson-Crick base-pairing one might expect these mutations to abrogate the intricate pseudo-triloop structure which lends itself as a highly specific recognition element. However, as in many triloops, the non-planar arrangement of the loop residues, and often also of the closing base-pair, allows for unconventional pairings between all four bases [54]. In addition, numerous different intraloop interactions can occur, as is the case for the HBV ε apical loop ([39] and PDB: 2IXY) and also in numerous triloop sequences found in other RNA 3D structures [55]. Conceivably then, formation of a pseudo-triloop structure may not be restricted to the genuine ε loop sequence. In particular our fully functional loop mutants may therefore be attractive candidates for future structural studies by NMR. Notably, ultra-deep sequencing revealed a low frequency of four UGU variants in patient-derived HBV sequences, including one identical to our U13C mutant [50] and one similar to U13,15C, except that U13 was preserved (corresponding to U15C in our nomenclature). Although ultra-deep sequencing may pick up nonfunctional sequences the near wild-type-like amounts and patterns of viral DNA seen for the U13C and the U13,15C mutants (Fig. 3) is in line with in vivo viability of these variants.

Interestingly, the loop mutants U13A and U13,15A displayed an intermediate DNA pattern between A1/A2 and A9/A10 mutants, i.e. some full-length products were formed but the bulk of signals accumulated further down in the gel, though not as low as for the partially defective A9/A10 variants (Fig. 3). This would be in line with a contribution of the loop to initiation site selection (see above).

## Conclusions

The interaction between HBV P protein and ε is key to virus replication and thus also an attractive target for intervention. However, in the absence of direct structural data on P protein, let alone active P protein - ε complexes, knowledge of this interaction is still limited. Our mutational analysis of different manifestations of the interaction, i.e. DNA synthesis, pgRNA encapsidation and in vitro protein-priming, substantially extends previous investigations. Given the dependence of RNA structure on sequence and the highly dynamic nature of the P protein - ε RNA interaction (see below), it is not surprising that our study revealed a whole range of phenotypes but no simple correlation allowing to predict how a specific mutant will behave. However, several trends were observed that should be valuable in designing further experiments, including attempts to target the P - ε interaction for therapeutic intervention. First, our identification of numerous mutations that had little impact on replication suggests a substantial sequence space for escape mutants. Second, our data give hints as to which subregions in ε tend have the largest (though not exclusive) impact on general P binding, priming efficiency, and possibly initiation site selection; to our knowledge, this is the first demonstration that these different functional aspects of the P - ε interaction can be genetically uncoupled, which also paves new ways towards more effective ε RNA decoys. Third, our data raise basic structural issues, especially the tolerance of the apical pseudo-triloop to even double mutations although the complex structure of this subelement should make a highly specific recognition element. These variant RNAs therefore lend themselves for further direct structural investigation.

Lastly, the diversity of phenotypes, from no impact at all to interference with distinctly different steps towards DNA synthesis, suggests that each step requires a distinct conformation of the RNA in the P protein complex, or in other words, that there are more conformational states than just non-productive binding (exemplified by in vitro P - ε binding without priming [34]) and a priming-competent interaction. Accordingly, individual nucleotides within ε may have dynamically altering contact patterns with P protein, each specific for a specific stage from initial binding over pgRNA encapsidation and priming until the eventual replacement of ε upon primer translocation to DR1*. Possibly the wild-type ε sequence provides the optimal solution to fully comply with these changing requirements.

## Materials and Methods

The sequences of the oligonucleotides used to introduce the indicated mutations into ε and for RT-PCR of β-actin mRNA are given in Table S1.

### Prediction for the 3D structure of ε

The 3D structure of ε was modeled using programs MC-Fold and MC-Sym [46], including experimental NMR data (PDB entry: 2IXY) of the apical stem-loop of ε [39].

### Plasmid constructs

The full-length HBV expressing vectors carrying mutant ε sequences were generated by site-directed mutagenesis. First, synthetic oligonucleotides (Table S1) carrying the desired nucleotide exchanges (Fig. 2B) were used as PCR templates, then the appropriately restricted PCR products were transferred into the Eco RV - Hind III sites of pCH-9/3091B, a previously constructed HBV vector carrying a deletion in the 5′-ε sequence [40], thus restoring an analogous arrangement as in the wild-type HBV expressing vector pCH-9/3091 [47]. The pcDNA3.1–3FlagP expression vector for HBV P protein with an N-terminal 3×FLAG tag was constructed by insertion of the P protein coding sequence into the Hind III/Xho I sites of pcDNA3.1(+) (Invitrogen), followed by insertion of the 3xFLAG coding sequence between the Nhe I and Hind III restriction sites. pcDNA3.1-ε, expressing the HBV ε sequence [44], was constructed by insertion of the wild-type ε sequence into the Kpn I/Apa I sites of pcDNA3.1(+). Vectors for mutant ε sequences were constructed by using pcDNA3.1-ε as PCR template for site-directed mutagenesis (QuikChange™ Site-Directed Mutagenesis Kit, Stratagene). All resulting constructs were confirmed by sequencing the relevant regions on the plasmids.

### Transfection and isolation of core particles

Transfections of HepG2 hepatoma cells with pCH-9/3091 and its derived constructs, and isolation of cytoplasmic core particles were performed as previously described [40]. Transfections of HEK293T cells with pcDNA3.1-3FlagP alone, or together with pcDNA3.1–ε or individual pcDNA3.1-ε mutants were performed exactly as described in [44].

### Southern blotting

To analyze HBV DNAs by Southern blotting, cytoplasmic core DNA was extracted, separated by agarose gel electrophoresis and, after blotting, hybridized to a ^32^P-labeled random-primed probe covering the whole HBV genome as previously described [40].

To directly analyze viral DNA content of nucleocapsids, intact cytoplasmic core particles were separated by 1% native agarose gel electrophoresis (NAGE) before blotting on nylon membranes by capillary transfer. The membranes were first soaked with denaturing buffer (0.2 M NaOH, 1.5 M NaCl) for 15 sec to release DNA from capsids, and then were neutralized by exposure to 0.2 M Tris/HCl (pH 7.5) supplemented with 1.5 M NaCl for 15 sec. After washing with distilled water, the membranes were cross-linked by UV irradiation and hybridized to a (+)-polarity HBV probe generated by nonsymmetrical PCR amplification of nucleoties 837–1284 of the HBV DNA, using Eco RI linearized plasmid pCH-9/3091 as template, a (+)-primer (5′-CTAGACACTATTTACACACT-3′) and dNTP mix containing α-^32^P-dATP (3,000 Ci/mmol).

### RNase protection assay (RPA)

To analyze encapsidated pgRNA, core pgRNA was extracted from an aliquot equivalent to four-fifth of the transfected cells from a 10 cm diameter plate as previously described [56]. To analyze cytoplasmic pgRNA, total RNA from the remainder one-fifth transfected cells of a 10 cm diameter plate was extracted with Trizol (Invitrogen). Both RNA fractions were then subjected to RNase protection assays using the RPA II kit (Ambion). The probe consisted of a 314 nucleotide antisense transcript comprising about 270 nucleotides complementary to HBV positions 3271–3282/1–261, and 41 nucleotides of non-matching polylinker sequence. All steps were performed as recommended by the manufacturer.

### Endogenous polymerase assay (EPA)

EPA was performed as previously described [40] by incubating cytoplasmic core particles at 37° C for 6h with α-^32^P-dCTP (3,000 Ci/mmol) or α-^32^P-dATP (3,000 Ci/mmol) supplemented with a mixture of the other three dNTPs. The reaction products were separated on a 1% agarose gel and then subjected to dry gel autoradiography.

### In vitro priming assay

Two days post-transfection, the FLAG-tagged P proteins, expressed alone or co-expressed with wild-type or mutant ε RNAs, were immobilized to anti-FLAG beads as described [44]. An aliquot equivalent to one-third of the purified complex was used for in vitro priming as reported, with minor modifications. Briefly, 38 µL TMgNK buffer (20 mM Tris/HCl [pH 7.0], 15 mM NaCl, 10 mM KCl, 4 mM MgCl_2_) supplemented with protease inhibitors (1×EDTA-free protease inhibitor cocktail and 1 mM PMSF), DTT (4 mM) and RNasin Plus RNase inhibitor (1 U/ µL) was added after the FLAG lysis buffer was removed from the bead-bound P protein. 1 or 2 µL α-^32^P-dATP (3,000 Ci/mmol) was then added. After incubating at 25° C for 4 h with shaking, the beads were washed with TNK buffer (20 mM Tris/HCl [pH 7.0], 15 mM NaCl, 10 mM KCl) supplemented with protease inhibitors and β-ME. Next the beads were boiled in sample buffer and the released components were analyzed by SDS-PAGE in 10% polyacrylamide gels and subsequent dry gel autoradiography.

### Western blotting

Western blotting for core protein and capsids was conducted as described in [40].

### Quantitative evaluations

Signal intensities from Southern blots, endogenous polymerase assays and RNase protection assays were determined by phosphorimaging using OptiQuant 5.0 software (Perkin Elmer), and related to the respective signals from wild-type HBV transfected cells as detailed in the legends of the corresponding figures. Data were derived from at least two, and usually three independent transfections.

## Supporting Information

Figure S1 **Limited impact of mutations at upper stem positions A1, A2, A9, A10, U13 and U15 on ε - φ base-pairing.**
(PDF)

Table S1 **Oligonucleotides used in this study.**
(PDF)
