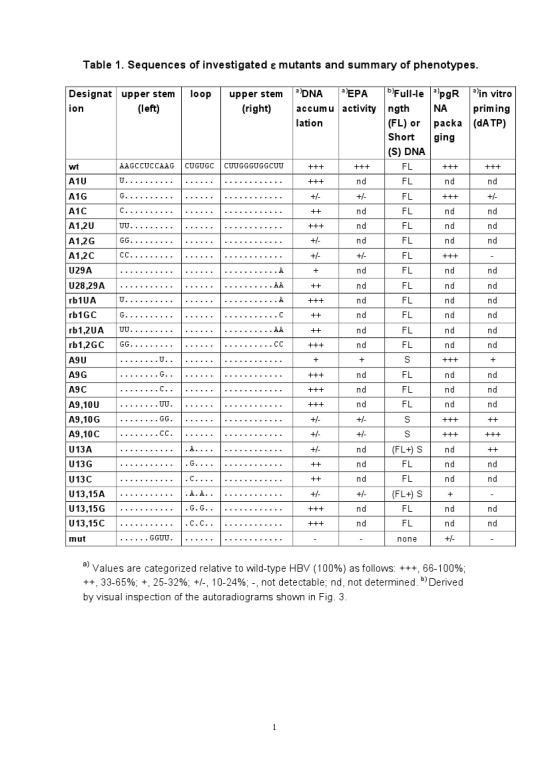# Correction: Evidence for Multiple Distinct Interactions between Hepatitis B Virus P Protein and Its Cognate RNA Encapsidation Signal during Initiation of Reverse Transcription

**DOI:** 10.1371/annotation/aa102795-3d0e-48b2-8898-5862c7d1e34b

**Published:** 2013-10-02

**Authors:** Hui Feng, Ping Chen, Fei Zhao, Michael Nassal, Kanghong Hu

There were errors in the layout of Table 1. A correct version of the table is available here: 

**Figure:**