# Supplementary material for: Evidence for Multiple Distinct Interactions between Hepatitis B Virus P Protein and Its Cognate RNA Encapsidation Signal during Initiation of Reverse Transcription
Source: PLoS One. 2013 Aug 20;8(8):e72798. doi: 10.1371/journal.pone.0072798 (PMC3748129; doi:10.1371/journal.pone.0072798)
Supplement: Figure S1 — Limited impact of mutations at upper stem positions A1, A2, A9, A10, U13 and U15 on ε - φ base-pairing. (PDF) [file pone.0072798.s001.pdf]

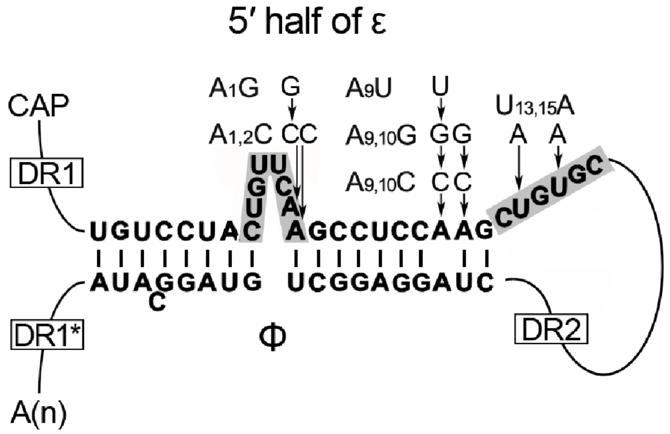


## Supplementary Fig. S1

**Limited impact of mutations at upper stem positions A1, A2, A9, A10, U13 and U15 on ε - ϕ base-pairing.** Base-pairing between the 5´ half of ε and the partly complementary downstream ϕ element as shown is proposed to contribute to efficient minus-strand DNA synthesis (1-3).The cartoon shows the nucleotide exchanges present in mutants A1G, A1,2C, A9U, A9,10G, A9,10C and U13,15A which all displayed reduced DNA accumulation in transfected cells. Some mutations have no impact (A1G, U13,15A) and some only a minor impact on the base-pairing pattern. Though not excluding a contribution of altered ε - ϕ pairing to reduced DNA accumulation, no strict correlation is obvious. Further clarification might be achieved by additional mutants in which ϕ is altered so as to exactly restore the native base-pairing pattern.

## Supplementary References:


1. Oropeza CE, McLachlan A (2007) Complementarity between epsilon and phi sequences in pregenomic RNA influences hepatitis B virus replication efficiency. Virology 359: 371-381.

2. Abraham TM, Loeb DD (2006) Base pairing between the 5’ half of epsilon and a cis-acting sequence, phi, makes a contribution to the synthesis of minus-strand DNA for human hepatitis B virus. J Virol 80: 4380-4387.

3. Abraham TM, Loeb DD (2007) The topology of hepatitis B virus pregenomic RNA promotes its replication. J Virol 81: 11577-11584.
