# Supplementary material for: Evidence for Multiple Distinct Interactions between Hepatitis B Virus P Protein and Its Cognate RNA Encapsidation Signal during Initiation of Reverse Transcription
Source: PLoS One. 2013 Aug 20;8(8):e72798. doi: 10.1371/journal.pone.0072798 (PMC3748129; doi:10.1371/journal.pone.0072798)
Supplement: Table S1 — Oligonucleotides used in this study. (PDF) [file pone.0072798.s002.pdf]

Supplementary Table S1. Oligonucleotides used in this study.

NAME	a) SEQUENCE (5´ to 3´)	Application
A1U	GTCCATGCCCCAAAGCCACCCAAGGCACAGCTTGGAGGCT**A**GAACAGTAGGACATGAAC	Template
A1G	GTCCATGCCCCAAAGCCACCCAAGGCACAGCTTGGAGGCT**C**GAACAGTAGGACATGAAC	"
A1C	GTCCATGCCCCAAAGCCACCCAAGGCACAGCTTGGAGGCT**G**GAACAGTAGGACATGAAC	"
A1,2U	GTCCATGCCCCAAAGCCACCCAAGGCACAGCTTGGAGGC**AA**GAACAGTAGGACATGAAC	"
A1,2G	GTCCATGCCCCAAAGCCACCCAAGGCACAGCTTGGAGGC**CC**GAACAGTAGGACATGAAC	"
A1,2C	GTCCATGCCCCAAAGCCACCCAAGGCACAGCTTGGAGGC**GG**GAACAGTAGGACATGAAC	"
U29A	GTCCATGCCCCA**T**AGCCACCCAAGGCACAGCTTGGAGGCTTGAACAGTAGGACATGAAC	"
U28,29A	GTCCATGCCCCA**TT**GCCACCCAAGGCACAGCTTGGAGGCTTGAACAGTAGGACATGAAC	"
rb1UA	GTCCATGCCCCA**T**AGCCACCCAAGGCACAGCTTGGAGGCT**A**GAACAGTAGGACATGAAC	"
rb1GC	GTCCATGCCCCA**G**AGCCACCCAAGGCACAGCTTGGAGGCT**C**GAACAGTAGGACATGAAC	"
rb1,2UA	GTCCATGCCCCA**TT**GCCACCCAAGGCACAGCTTGGAGGC**AA**GAACAGTAGGACATGAAC	"
rb1,2GC	GTCCATGCCCCA**GG**GCCACCCAAGGCACAGCTTGGAGGC**CC**GAACAGTAGGACATGAAC	"
A9U	GTCCATGCCCCAAAGCCACCCAAGGCACAGCT**A**GGAGGCTTGAACAGTAGGACATGAAC	"
A9G	GTCCATGCCCCAAAGCCACCCAAGGCACAGCT**C**GGAGGCTTGAACAGTAGGACATGAAC	"
A9C	GTCCATGCCCCAAAGCCACCCAAGGCACAGCT**G**GGAGGCTTGAACAGTAGGACATGAAC	"
A9,10U	GTCCATGCCCCAAAGCCACCCAAGGCACAGC**AA**GGAGGCTTGAACAGTAGGACATGAAC	"
A9,10G	GTCCATGCCCCAAAGCCACCCAAGGCACAGC**CC**GGAGGCTTGAACAGTAGGACATGAAC	"
A9,10C	GTCCATGCCCCAAAGCCACCCAAGGCACAGC**GG**GGAGGCTTGAACAGTAGGACATGAAC	"
U13A	GTCCATGCCCCAAAGCCACCCAAGGCAC**T**GCTTGGAGGCTTGAACAGTAGGACATGAAC	"
U13G	GTCCATGCCCCAAAGCCACCCAAGGCAC**C**GCTTGGAGGCTTGAACAGTAGGACATGAAC	"
U13C	GTCCATGCCCCAAAGCCACCCAAGGCAC**G**GCTTGGAGGCTTGAACAGTAGGACATGAAC	"
U13,15A	GTCCATGCCCCAAAGCCACCCAAGGC**T**C**T**GCTTGGAGGCTTGAACAGTAGGACATGAAC	"
U13,15G	GTCCATGCCCCAAAGCCACCCAAGGC**C**C**C**GCTTGGAGGCTTGAACAGTAGGACATGAAC	"
U13,15C	GTCCATGCCCCAAAGCCACCCAAGGC**G**C**G**GCTTGGAGGCTTGAACAGTAGGACATGAAC	"
mut	GTCCATGCCCCAAAGCCACCCAAGGCGCGGC**AACC**AGGCTTGAACAGTAGGACATGAAC	"
eps-for	CTGCGATATCATCTCTTGTTCATGTCCTAC	PCR
eps-rev	TTCTTTATAAGCTTCGATGTCCATGCCCCA	PCR
b-act-for	CACTGTGCCCATCTACGAG	RT-PCR
b-act-rev	CCATCTCCTGCTCGAAGTC	RT-PCR

a) The template oligonucleotides had anti-sense orientation; mutated residues are shown in bold face. PCR primers eps-for and eps-rev were used for amplification and the products were restricted with Eco RV and Hind III (recognition sites underlined in the sequences) for cloning.
